# Right Hemisphere Grey Matter Volume and Language Functions in Stroke Aphasia

**DOI:** 10.1155/2017/5601509

**Published:** 2017-05-09

**Authors:** Sladjana Lukic, Elena Barbieri, Xue Wang, David Caplan, Swathi Kiran, Brenda Rapp, Todd B. Parrish, Cynthia K. Thompson

**Affiliations:** ^1^Center for the Neurobiology of Language Recovery, Northwestern University, Evanston, IL, USA; ^2^Department of Communication Sciences and Disorders, School of Communication, Northwestern University, Evanston, IL, USA; ^3^Department of Radiology, Feinberg School of Medicine, Northwestern University, Chicago, IL, USA; ^4^Department of Neurology, Massachusetts General Hospital, Harvard Medical School, Boston, MA, USA; ^5^Department of Speech, Language, and Hearing, College of Health & Rehabilitation, Boston University, Boston, MA, USA; ^6^Department of Cognitive Science, Krieger School of Arts & Sciences, Johns Hopkins University, Baltimore, MD, USA; ^7^Department of Neurology, Neurology, Feinberg School of Medicine, Northwestern University, Chicago, IL, USA

## Abstract

The role of the right hemisphere (RH) in recovery from aphasia is incompletely understood. The present study quantified RH grey matter (GM) volume in individuals with chronic stroke-induced aphasia and cognitively healthy people using voxel-based morphometry. We compared group differences in GM volume in the entire RH and in RH regions-of-interest. Given that lesion site is a critical source of heterogeneity associated with poststroke language ability, we used voxel-based lesion symptom mapping (VLSM) to examine the relation between lesion site and language performance in the aphasic participants. Finally, using results derived from the VLSM as a covariate, we evaluated the relation between GM volume in the RH and language ability across domains, including comprehension and production processes both at the word and sentence levels and across spoken and written modalities. Between-subject comparisons showed that GM volume in the RH SMA was reduced in the aphasic group compared to the healthy controls. We also found that, for the aphasic group, increased RH volume in the MTG and the SMA was associated with better language comprehension and production scores, respectively. These data suggest that the RH may support functions previously performed by LH regions and have important implications for understanding poststroke reorganization.

## 1. Introduction

Research shows that undamaged tissue in both the contralesional (usually right) and ipsilesional (left) hemispheres of the brain is recruited to support recovery in stroke-induced aphasia (see reviews by [1–7]). Neuroimaging studies show that in early stages of recovery, the right hemisphere (RH) is active during language tasks; however, a shift in activation to the left hemisphere (LH) regions has been found across tasks, including word repetition, rhyme judgment, auditory word/sentence comprehension, semantic association, and reading [8–12]. Functional neuroimaging studies conducted with chronic aphasic individuals also confirm a primary role of ipsilesional tissue in recovery, finding significant correlations between recovery of language function and activation in the LH during confrontation-naming tasks [13, 14].

Other studies, however, have found RH recruitment, even in late stages of recovery [15–23]. Patients studied by Musso and coworkers [18] with lesions in the LH superior temporal gyrus (STG) showed activation in the RH STG during a sentence comprehension task, which positively correlated with off-line performance on a measure of auditory verbal comprehension. Similarly, Perani et al. [20] reported patients with damage to the LH inferior frontal gyrus (IFG) who showed activation of the RH homologue of this region when performing a verbal fluency task. In keeping with these findings, a recent meta-analysis of 12 neuroimaging studies in chronic stroke-induced aphasia [24] showed that, although aphasic individuals evince activation in the LH (i.e., the IFG and middle temporal gyrus (MTG), similar to healthy controls, as well as the left middle frontal gyrus (MFG) and insula), they also show the right hemisphere activation across a variety of language tasks (i.e., in the postcentral gyrus (PCG) and MTG).

Evidence of RH recruitment to support language recovery also comes from studies examining treatment-induced neural plasticity in chronic aphasia, showing increased RH activation associated with treatment gains [17, 25–31]. Recently, Kiran et al. [29] examined neural activation and effective connectivity within the left language network and right homologous regions following language treatment in eight chronic aphasic individuals. The results showed posttreatment increases in neural activity, bilaterally, in picture naming and semantic feature verification tasks. Importantly, effective connectivity maps in individuals with aphasia revealed that the LH IFG and the connection between the RH IFG and the RH MFG, respectively, most consistently modulated as a function of rehabilitation. Several other studies have shown similar patterns of posttreatment increases in the RH regions on picture naming (see [13, 32]) as well as semantic (compared to orthographic and phonological) processing tasks [33, 34]. Thompson et al. [35] also found a bilateral posttreatment upregulation of activation in the temporoparietal region in six chronic aphasic individuals who showed treatment-induced improvement in syntactic processing. These data indicate that the RH regions are engaged in language processing following damage to LH language networks. However, whether or if engagement of the RH is associated with maximally effective language processing has been questioned.

Some research suggests that rather than benefitting language processing, RH recruitment may be maladaptive and reflect inefficient language processing, finding, for example, either no association between increased RH activation and performance on a verb generation task [36] or a correlation between RH frontal activation and production of inaccurate responses on a picture-naming task [37]. An inefficient/maladaptive role of the RH has also been suggested by brain stimulation studies, showing that inhibitory repetitive transcranial magnetic stimulation (rTMS) applied to the RH regions (i.e., the IFG) improves language function ([38–41]; also see [6] for review), putatively secondary to inhibition of the maladaptive RH regions, which thereby facilitates LH processing (but see [42–44] for evidence suggesting that excitatory stimulation directed to the RH positively impacts language performance in chronic aphasic individuals). These and other studies have led to the assertion that recruitment of ipsilesional, rather than contralesional, tissue into the language network may result in greater language gains. Some recent neuroimaging studies also suggest that the contribution of the RH to recovery from aphasia may not reflect restoration of language processes, but rather the engagement of domain-general networks responsible for attention and cognitive control [45, 46], or processing of perceptual aspects of verbal stimuli [47].

One way to estimate the functionality of cortical tissue is to examine the density of grey matter (GM) tissue, with the assumption that greater GM volume is associated with greater functionality and lesser (i.e., cortical atrophy) associated with decreased function [48, 49]. Studies on the recovery of motor function in chronic stroke have found both increases and decreases in GM volume in motor regions of the brain in patients following recovery (versus healthy controls). Zhang et al. [50] examined 26 hemiparetic individuals (with partial or complete recovery) and 25 age-matched controls on motor tasks before and after physical therapy. They found reduced cortical volume in the ipsilesional motor region for all patients compared to controls with no GM changes in contralesional motor areas. However, in another study, Gauthier et al. [51] found increased GM volume in RH motor regions, homologous to lesioned tissue in the LH, associated with recovery of function in 85 individuals with chronic stroke (also see [52]).

Few studies have examined GM volume in patients with cognitive impairments resulting from stroke. Stebbins et al. [53], using voxel-based morphometry (VBM, [54]), reported significant GM volume reductions (mostly in the thalamus) for stroke patients (*n* = 91) with cognitive impairment (compared to those without). In another study, Xing et al. [55] reported increased GM volume (compared to healthy, unimpaired control participants) in the right temporoparietal cortex (i.e., the supramarginal gyrus (SMG) and STG) in individuals with chronic stroke-induced aphasia. They further showed that GM volume was positively associated with overall aphasia severity as well as performance on production subtests of the *Western Aphasia Battery-Revised* (WAB-R; [56]) (i.e., spontaneous speech, repetition, and naming). Although the study was not longitudinal, the authors interpreted the results as suggesting a compensatory role of the right posterior regions in chronic aphasia. In addition, by partialing out participant variables (e.g., age, gender, level of education, and handedness) as well as the effect of lesion volume on language performance, the authors found the right hypertrophic temporoparietal regions, suggesting that these regions play a role in language recovery.

The present study examined RH GM volume in individuals with chronic stroke-induced aphasia and cognitively healthy people using voxel-based morphometry (VBM; [54]), a voxel-wise neuroimaging technique used for measuring variables associated with brain anatomy (e.g., GM volume). We compared group differences (healthy versus aphasic participants) in GM volume in the entire RH and in RH regions-of-interest (ROIs) where aphasic individuals exhibited a significant relation between GM volume and language performance. Given that lesion site is a critical source of heterogeneity associated with poststroke language ability, we then used voxel-based lesion symptom mapping (VLSM; [55]) to examine the relation between lesion site and language performance in the aphasic participants. Finally, using results derived from the VLSM analysis as a covariate (following Xing et al. [56]), we evaluated the relation between GM volume in the RH and language scores across domains, including comprehension and production processes both at the word and sentence level and across spoken and written modalities.

In line with the aforementioned studies showing structural changes after LH stroke, we expected differences in GM volume in the RH in the aphasic participants compared to healthy controls (i.e., either decreased or increased volumes). We also predicted that if the RH supports language function, then a positive correlation between performance on language tasks and RH GM volume would be observed, independently of differences in lesion volume. Conversely, if the RH does not support language functions, we expected no correlation between language performance and RH GM volume in the group of aphasic participants.

## 2. Method

### 2.1. Participants

Forty participants with aphasia (14 female) resulting from a single-left hemisphere stroke and 40 cognitively healthy age-matched (AM) controls (18 female) were recruited for the study from three research sites: Northwestern (NU), Boston (BU and MGH), and Johns Hopkins (JHU) Universities. All were native English speakers, passed a pure-tone audiometric screening and evinced normal or corrected-to-normal vision (self-reported). All participants were right handed, with the exception of one aphasic speaker who was left handed prior to the stroke that affected his left hemisphere. Participants at each site were recruited as part of a large-scale study examining treatment-induced changes in brain function and, hence, were selected for specific language-deficit patterns: agrammatism (NU), anomia (BU, MGH), and dysgraphia (JHU).

Across sites, the aphasic and control groups were matched for age (*t* (77.9) = −0.166; *p* > 0.05), ranging from 35 to 81 (59.4 ± 12.4 yrs) and 24–80 (58.9 ± 11.8 yrs) for the two participant groups, respectively, and years of education (aphasic group mean = 16.1 ± 2.2; control group mean = 15.6 ± 2.4 (*t* (71.5) = −0.936; *p* > 0.05)). Within site, participant groups also did not differ in age (NU: *t* (20) = 1.678, *p* > 0.05; BU: *t* (31.7) = −0.882, *p* > 0.05; and JHU: *t* (21.8) = −0.293, *p* > 0.05), and years of education were matched between participant groups for all sites except JHU, where patients were more highly educated than the control participants (NU: *t* (19) = −0.571, *p* > 0.05; BU: *t* (22.7) = 0.398, *p* > 0.05; and JHU: *t* (18.9) = −2.275, *p* = 0.035). All participants completed written consent form approved by NU, BU, and JHU Institutional Review Boards (IRB). See Table 1 for demographic data.

Aphasic participants were at least eight months post onset of stroke (57.2 ± 52.3 months) and presented with aphasia based on administration of the *Western Aphasia Battery-Revised* (WAB-R; [57]) and a uniform set of cross-site language measures. The WAB Aphasia Quotient score (WAB-AQ) ranged from 25.2 to 98.4 (70.2 ± 20.5), with no significant differences between participants enrolled at NU and those enrolled at the other sites (NU versus BU: *t* = 1.282, *p* > 0.05; NU versus JHU: *t* = −1.536, *p* > 0.05), while aphasic participants enrolled at BU showed lower WAB-AQ scores than those at JHU (*t* = −2.452, *p* = 0.021). The type and severity of language impairment were characterized using a test battery, which included selected subtests of the *Northwestern Naming Battery* (NNB; [58]), *Psycholinguistic Assessments of Language Processing in Aphasia* (PALPA; [59]); and *Northwestern Assessment of Verbs and Sentences* (NAVS; [60]).

### 2.2. Language Measures

Language measures selected to examine participants' abilities across domains included the confrontation-naming (CN) and auditory comprehension (AC) subtests from the NNB to quantify single-word naming and comprehension. These subtests use the same sets of nouns and verbs for testing in both domains. From the PALPA, subtests 35, 40, and 51 were selected to evaluate oral reading of words with regular and irregular orthography (PALPA35), spelling-to-dictation of words with high and low frequency (PALPA40), and semantic association between written words (PALPA51), respectively. Finally, the Sentence Production Priming Test (SPPT) and the Sentence Comprehension Test (SCT) from the NAVS were used to evaluate production and comprehension of sentences of different complexity (same sentences tested across domains).

### 2.3. MRI Image Acquisition

A 3T Trio Siemens scanner at NU, a 3T Skyra at BU, and a Phillips Intera scanner at JHU were used to obtain anatomical T1-weighted scans. Across all sites, standard T1-weighted 3D MPRAGE scans were acquired in the sagittal plane (TR/TE = 2300/2.91 ms, Flip angle = 9°, 1 × 1 × 1 mm), together with a T2-weighted FLAIR sequence (TR/TE = 9000/90 ms, Flip angle = 150°, 0.86 × 0.86 × 5 mm), which was coregistered and resliced for resolution and orientation consistency with T1 images by participant. Prior to the study, imaging sequences were equated across sites, with the same parameters used for data acquisition across scanners, and quality control was performed to ensure high-quality data from each site.

### 2.4. MRI Preprocessing (NUNDA Pipeline Description)

Anatomical images were corrected for bias field inhomogeneities [61], and lesioned brain regions were masked out before being subjected to a standard voxel-based morphometry workflow using VBM8 toolbox (developed by Christian Gaser). Analysis steps included tissue segmentation, rigid registration, and DARTEL normalization to the template space (Template_1_IXI550_MNI152.nii). The normalized and modulated GM segments were smoothed by 8 mm FWHM Gaussian Kernel and masked using a right hemisphere (RH) GM mask of the T1 brain template.

### 2.5. Lesion Identification

**Figure 1:**
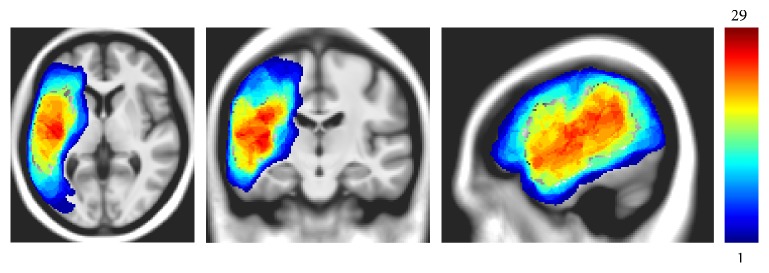
Lesion overlap map of 40 participants with aphasia, showing areas of overlap, from no overlap (blue) to maximum overlap (red; *N* = 29 participants).

The chronic stroke lesion mask was manually generated using MRIcron [62] in native space by trained professionals from each site. To delineate the borders of the necrotic tissue for each patient, intensity measures for white and grey matter (WM and GM) in the contralateral right hemisphere were used for each axial slice. The left hemisphere lesioned tissue was drawn on each slice using the pen tool of MRIcron, and then applying the minimum intensity to the outlined area using the intensity filter function. Additional manual correction was applied by visualizing the volume in all three planes simultaneously. All brains and lesions were normalized into Montreal Neurological Institute (MNI) space as part of the anatomical preprocessing pipeline provided by the Northwestern University Neuroimaging Data Archive (NUNDA; [63]) prior to VLSM analysis.  Figure 1 displays a lesion overlap map for the aphasic participant group.

### 2.6. Data Analyses

#### 2.6.1. Analysis 1: Between-Subject (Aphasic Participants, AM Controls) Differences in GM Volume

**Figure 2:**
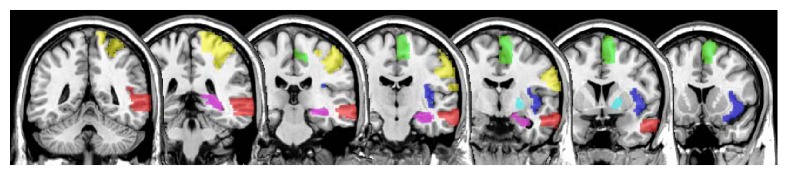
Six right hemisphere regions of interest (ROIs), derived from VBM analysis, used to evaluate between-group differences in the grey matter volume. SMA = green, MTG = red, insula = blue, hippocampus = violet, postcentral = yellow, and pallidum = cyan.

The group differences (healthy versus aphasic participants) in grey matter volume were examined in the entire RH and in selected region of interest (ROI). The ROIs were derived from the VBM Analysis 3 (see next). Specifically, for any cluster in which grey matter volume was found to be significantly associated with any of our seven language measures (VBM Analysis 3), we identified the ROI within which the peak voxel for that cluster resided. The so identified ROIs included the right supplementary motor area (SMA), MTG, insula, hippocampus, postcentral, and pallidum areas (see Figure 2()). These ROIs were anatomically defined using the AAL atlas within the MarsBaR toolbox in SPM8 [64]. For each ROI, a linear regression analysis was conducted using R 3.2.3 [65], where the mean GM volume was used as a dependent variable, and group (healthy versus aphasic individuals) as an independent variable. Age and total intracranial volume (computed as the sum of grey and white matter and cerebrospinal fluid) were included as covariates in all regression models. Additionally, *p* values resulting from regression analyses were corrected for the number of ROIs examined using the Benjamini-Hochberg correction [66], with *n* being the total number of ROIs examined (6). Only Benjamini-Hochberg-corrected results are reported in the Results.

#### 2.6.2. Analysis 2: The Effect of LH Lesion on Language Performance

A voxel-based lesion symptom mapping (VLSM) approach was used to analyze the relationship between lesions in the left hemisphere and language performance [55], using the VLSM toolbox (http://www.crl.ucsd.edu/vlsm) running under Matlab R2014a. (MathWorks Inc., 2014). The participants' lesion images (binary) and language scores (% correct) were entered into a VLSM analysis. For each voxel, aphasic participants were divided into two groups based on the presence (1) or absence (0) of a lesion in that voxel. Only voxels in which more than four (at least 10%) participants had lesions were included in the analysis. VLSM analyses were run with *n* = 1000 permutation tests, resulting in T-maps that reflected critical regions in the LH where lesioned tissue was associated with performance on a given language measure. The total lesion volume was automatically calculated from the lesion masks and served as a covariate in the analysis. Significant results were derived from voxel-wise *t*-tests using a threshold of *p* < 0.05 with permutation-based correction for multiple comparisons. Cluster level *p* values then underwent the Benjamini-Hochberg correction [66] for multiple comparisons (with *n* being the total number of VLSM analyses conducted, that is, seven, one for each language measure). Only corrected *p* values are reported in the text. Additionally, effect sizes for significant comparisons were calculated using the following formula, based on the T-statistics (t) and the degrees of freedom (df) $\sqrt[{2}]{\left(t^{2}/\left(t^{2}+df\right)\right)}$.

#### 2.6.3. Analysis 3: The Effect of RH GM Volume on Language Performance

The relationship between grey matter volume in the right hemisphere and language performance on the seven language domain measures was analyzed by performing voxel-wise multiple linear regression using the VBM8 toolbox (http://dbm.neuro.uni-jena.de/vbm8) in Statistical Parametric Mapping software (SPM8; http://www.fil.ion.ucl.ac.uk/spm). The segmented, modulated, normalized, and smoothed GM images and language scores (% correct) were entered in each regression model, resulting in T-maps that showed regions where GM volume was significantly associated with language performance. As pointed by Xing et al. [56], when determining the contribution of GM volume in the RH to language performance, it is important to account for the contribution of LH lesioned tissue to the performance on the same language measure, as any correlation found between RH GM volume and language performance may be influenced by the effect of the LH lesion size/site on the participants' performance. In order to account for this, as in Xing et al. [56], the “proportion of critical area of damage” (PCAD) was entered as a covariate together with age and the total intracranial volume (computed as the sum of grey and white matter, and cerebrospinal fluid) in the VBM analysis to partial out their effects on language performance. The PCAD was computed by intersecting the map derived from the group VLSM with each participant's lesion, divided by the VLSM map volume. The PCAD, then, ranged from 0 (when there was no overlap between a patient's lesion and the group map) to 1 (when there was total overlap, with all voxels lesioned in the group map also lesion for the patient). Group T-maps derived from VBM analyses conducted on language measures were then thresholded by determining the minimum cluster size based on a *p* < 0.001 voxel-level threshold and on an estimate of image smoothness in AFNI [67], following the evidence of a disproportionately high rate of false-positive results yielded by family-wise (FWE) cluster-level correction in SPM [68]. The group residuals derived from the SPM T-maps were run through the 3dfwhmx function in AFNI, which uses the latest version of the autocorrelation function, to derive an estimate of image smoothness, and thresholded at a conservative *p* < 0.001 voxel level using the 3dClustSim function, to determine the appropriate cluster size threshold for each regression analysis. T-maps were also multiplied by a GM mask to ensure significant clusters would be restricted to grey matter and by the Automated Anatomical Labeling (AAL) atlas to obtain MNI coordinates for every peak in every significant cluster. The AAL template was then overlaid onto each binarized T-map using MRIcron [62] to identify the region corresponding to each peak coordinate. Cluster *p* values were finally corrected for multiple comparisons (with *n* being the number of regressions performed, that is, seven, one for each language measure) using the Benjamini-Hochberg correction [66]. As for VLSM, effect sizes for each VBM regression analysis were computed as described above, and only corrected *p* values are reported in the text.

## 3. Results

### 3.1. Language Measures

Participant scores derived from administration of language measures across language domains are shown in Table 2. Within the comprehension domain, participants performed well on spoken word comprehension (NNB AC: 92.5 ± 14.5), while scores obtained on semantic association and sentence comprehension were lower on average and more variable (PALPA51: 64.0 ± 20.1; NAVS SCT: 71.4 ± 17.3). Within the production domain, aphasic participants scored better on spoken word production and oral reading (NNB CN: 70.5 ± 29.7; PALPA 35: 65.1 ± 34.3) than on spelling-to-dictation (PALPA40: 37.1 ± 26.8) and sentence production (NAVS SPPT: 39.4 ± 31.5).

### 3.2. Between-Subject (Aphasic Participants, AM Controls) Differences in GM Volume

Between-subject analysis of GM volume for the entire RH showed no significant differences between the aphasic participants and age-matched controls. The results of the ROI analyses revealed between-group differences in the right SMA (*p* = 0.054), where patients showed reduced GM volume compared to healthy participants. To follow up on this result, a median split was used to divide patients into two groups, that is, those with good (>65% correct) (*n* = 21) and poor (<65% correct) (*n* = 19) production ability, based on a composite score (the average percentage correct across the three production measures: spoken word production, oral reading, and sentence production). A between-group (healthy controls, good performers, and poor performers) analysis was run on the mean RH GM volume in the SMA, with age and total intracranial volume included as covariates. The results showed a significant difference between healthy controls and poor performers in GM volume within the RH SMA (*p* = 0.004), while no difference was found between healthy controls and good performers (*p* = 0.294).

### 3.3. Effect of the LH Lesion on Language Performance (VLSM Results) in Aphasic Participants

**Figure 3:**
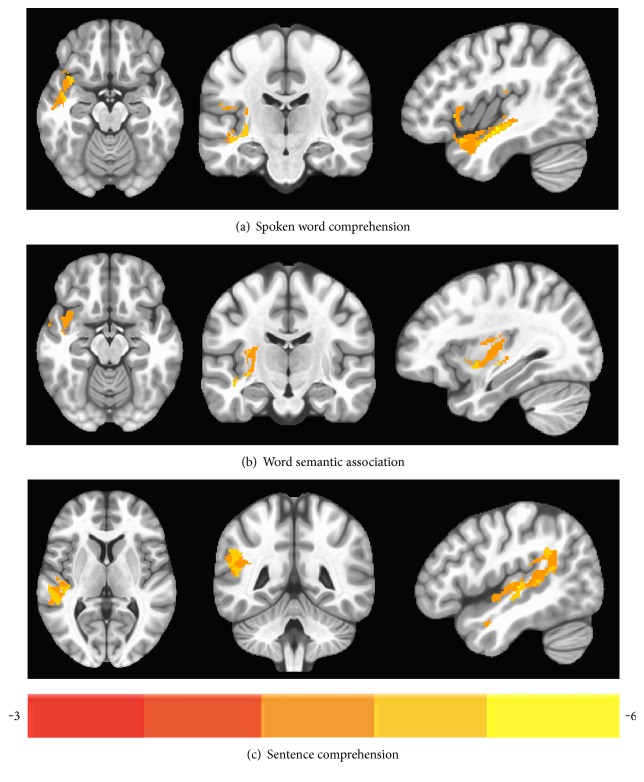
VLSM maps showing left hemisphere regions that were significantly associated with language performance. Panels (a–c) display lesions correlated with comprehension measures: (a) spoken word comprehension, (b) word semantic association, and (c) sentence comprehension. All voxels shown in color survived a threshold of *p* < 0.05, based on cluster size and the permutation method. The color bar reflects the range of *t* values from minimum (red) to maximum (yellow).

The following results were derived from the VLSM analysis and illustrate the relation between LH lesion and language performance. The results of VLSM analyses are reported in Table 3.  Figure 3 displays the relationship between LH lesion site and performance on each language measure.

For measures assessing comprehension, VLSM analysis of *spoken word comprehension* revealed a trend toward a negative relationship between lesions in the left IFG, STG, putamen, and rolandic operculum and spoken word comprehension scores (*p* = 0.068). Similarly, *word semantic association* performance was negatively associated with lesions in the left IFG, STG, and putamen, as well as in two unlabeled clusters spatially contiguous to the insula and caudate (*p* = 0.068). Finally, for *sentence comprehension*, a trend toward a negative relationship was observed with lesions in the left MTG and STG (*p* = 0.068).

VLSM analyses of production measures revealed no significant relationships between lesions and performance on *spoken word production*, *oral reading*, *spelling*-*to*-*dictation*, or *sentence production* (all corrected ps > 0.1).

### 3.4. Effect of the RH GM Volume on Language Performance (VBM Results) in Aphasic Participants

**Figure 4:**
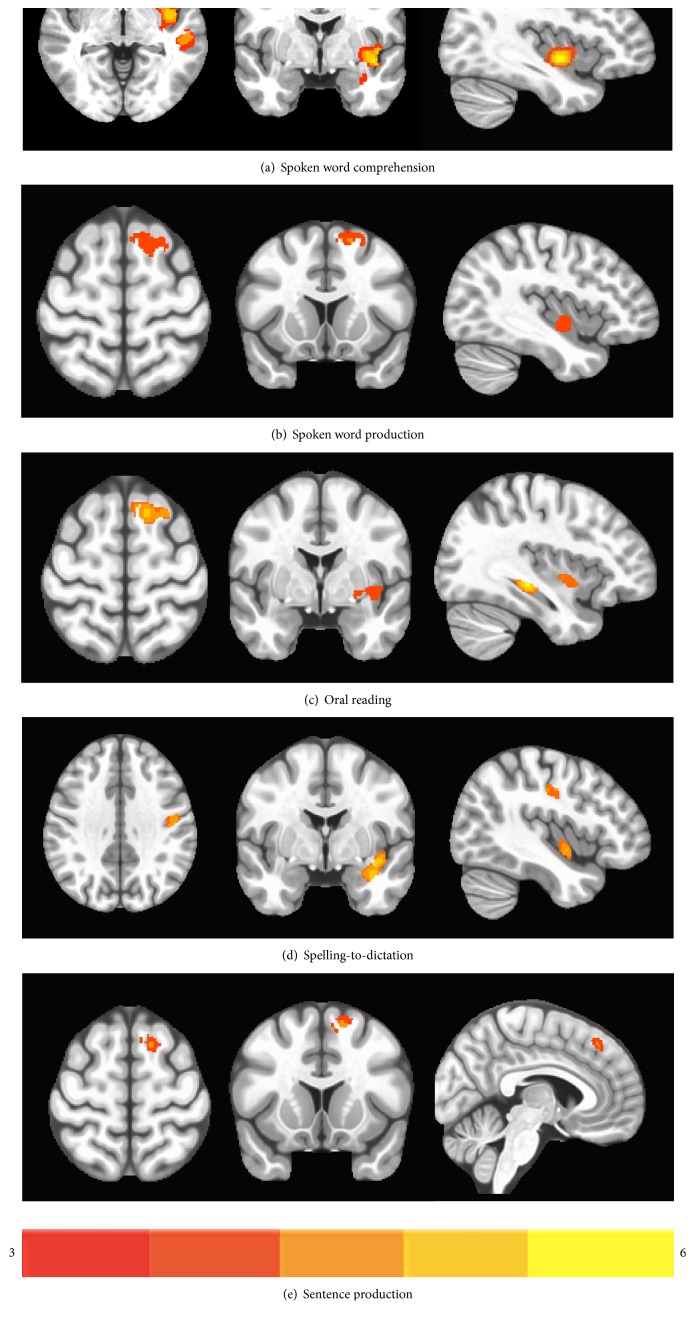
VBM maps showing right hemisphere regions where GM volume was significantly associated with language performance. Panel (a) shows the relationship between RH gray matter volume and spoken word comprehension. Panels (b–e) display the relationship between RH gray matter volume and production measures: (b) spoken word production, (c) oral reading, (d) spelling-to-dictation, and (e) sentence production. All voxels shown in color survived a threshold of *p* < 0.05, cluster-level FWE corrected. The color bar reflects the range of *t* values from minimum (red) to maximum (yellow).

The following results were derived from the VBM regression analysis and illustrate the relation between RH GM volume and language performance where the relations between LH lesioned tissue and language performance (as derived from the VLSM analyses) were taken into account and entered as nuisance variables. VBM maps indicating RH regions in which GM volume was significantly positively associated with language performance are shown in Figure 4. The results of VBM analysis for the aphasic participants are reported in Table 4.

The voxel-wise linear regression of *spoken word comprehension* on GM volume revealed a significant positive relationship between single-word comprehension scores and GM volume in the right MTG and insula. VBM analyses conducted on measures of *word semantic association* and *sentence comprehension* did not yield any significant clusters. For production measures, the voxel-wise linear regression of *spoken word production* on the GM volume revealed a significant positive relationship between word production scores and GM volume in the right SMA and insula. Similarly, *oral reading* and *sentence production* performances were positively related with GM volume within the right SMA, whereas *oral reading* performance was also associated with GM volume in the pallidum and hippocampus. Finally, for *spelling-to-dictation,* a positive relationship between GM volume and performance was observed in the right hippocampus and postcentral region.

## 4. Discussion

This study examined the right hemisphere (RH) grey matter (GM) volume in a group of 40 individuals with stroke-induced chronic aphasia using voxel-based morphometry (VBM). We first compared values derived from the patient group to those derived from 40 age-matched healthy controls, finding reduced GM volume in the RH supplementary motor area (SMA) in aphasic individuals compared to healthy age-matched controls. Follow-up analyses also revealed a significant difference in SMA GM volume only between healthy controls and aphasic individuals with more severe impairment in language production, while no difference emerged between patients with milder language production deficits and healthy individuals. Next, we evaluated the relation between RH GM volume and language performance in the aphasic participant group, controlling for the left hemisphere lesion site, using VBM. The results revealed two findings: (1) better word comprehension was associated with increased RH GM volume in the middle temporal gyrus (MTG) and insula, and (2) better word and sentence production was associated with increased RH GM volume in the SMA.

Language comprehension was evaluated using standardized measures of spoken word comprehension, semantic association, and sentence comprehension. The spoken word comprehension measure examined participants' ability to comprehend single words (nouns and verbs) from an array of semantically or argument structure-related items, respectively. Accordingly, failure on this task reflects inability to either link spoken words to objects/actions or to access semantic knowledge [58]. The semantic association task also examined word comprehension, although from the visual modality, requiring participants to select semantically related words. To perform the sentence comprehension (i.e., sentence-picture matching) task, individuals needed to access lexical and semantic information stored in long-term memory and integrate it into syntactic structure.

The VBM analysis shows that GM volume in the right MTG and insula was positively associated with performance on *spoken word comprehension,* but no association was found between the RH GM volume and the other two comprehension measures, that is, *semantic association* and *sentence comprehension* in any region. Lesion-deficit patterns derived from VLSM showed that lower performance on both word comprehension and semantic association measures were associated with a lesion in the left IFG and STG, whereas lower sentence comprehension scores were associated with lesions in the left STG and MTG. However, given that VLSM analyses yielded results that were only marginally significant after applying a correction for multiple comparisons, the discussion will focus primarily on the results of the VBM analyses, and VLSM results will be discussed within the context of the explanation of the VBM results.

The finding of an association between performance on spoken word comprehension and GM volume in the right temporal cortex suggests that the RH temporal region may support lexical access during word processing. This finding is in line with neuroimaging studies showing increased RH activation in temporal lobe regions with improved lexical-semantic (compared to orthographic and phonological) processing in aphasic participants [33]. The results are also consistent with studies showing increased posttreatment activation bilaterally in the MTG (in addition to the frontal cortex) on a semantic feature verification task, which also requires access to semantic knowledge [24, 29]. Moreover, when looking at the results of the lesion-deficit analyses for spoken word comprehension and semantic association tasks, within the context of the aforementioned RH results, damage in the left IFG and STG likely affected lexical selection and storage of lexical representations, respectively. This is consistent with studies showing an association between damage to temporal lobe structures and comprehension/semantic deficits in aphasic individuals [69, 70].

In addition to recruitment of the RH temporal lobe, the VBM analysis showed that performance on spoken word comprehension was also associated with GM volume in the insula. Neuroimaging studies have shown activation in the left insula during phonological discrimination tasks [71, 72], although its role in word processing is debated, as several neuroimaging studies have found activation of the insula using a variety of language tasks including naming and word generation ([73–75], see [76] for a review). However, a role for the insula in word comprehension has been suggested in functional connectivity studies, showing significant connections between the insula and the temporal lobe, namely the STG and MTG [76].

Notably, we observed no relationship between GM volumes and sentence comprehension in the right hemisphere. According to most studies with cognitively healthy people, sentence comprehension is supported by a primarily left lateralized temporofrontal network (see [77] for a neurocognitive model of sentence comprehension; also see [5]), with neuroimaging studies showing increased activation in the left frontal and posterior temporal cortex when comparing sentences with plausible versus implausible meanings [78], grammatical versus ungrammatical sentences [79], or syntactically complex versus simple sentences [31, 80, 81]. These findings suggest that the left temporal and frontal tissue is recruited when strategic, combinatorial, and/or memory processes come into play during sentence processing [82]. In the present study, the absence of a sentence-level comprehension effect in the RH as well as our VLSM lesion-deficit results, revealing a significant negative correlation in the left STG and MTG and sentence comprehension, that is, poorer sentence comprehension was associated with lesions in these regions, consistent with previous findings [31, 70, 83, 84], reflect a reliance on the left hemisphere for sentence comprehension for our patients [1].

Turning to language production, spoken word and sentence production, oral reading, and spelling-to-dictation were tested using standardized measures. Language production engages many of the same processes involved in comprehension, including semantic mediation, phonological processing, and in the case of sentence production, integration of semantic, and syntactic information. However, production also engages motor planning, articulatory, and associated processes.

For *spoken word production*, *sentence production*, and *oral reading*, we found increased RH GM volume in the right SMA associated with better performance. This finding is in line with the results of several neuroimaging studies, which have found significant SMA activation in production tasks in healthy speakers in both silent (covert) and overt production tasks (see [85] and [3] for review; [86–88]). Although SMA activation often is associated with motor planning and articulatory processes, some authors suggest that this region also is involved in lexical selection and word form encoding [89, 90]. Positive correlations between GM volume in the RH SMA found in the present study across production (but not comprehension) tasks support this, suggesting that the right homologue of the SMA may be recruited to support production processes in individuals with aphasia resulting from stroke.

In addition, the VBM analysis showed that performance on word production was associated with increased GM volume in the insula. Neuroimaging studies examining naming and word generation in healthy speakers have found LH insula activation (see [76] for a review). Previous lesion-deficit correlation analyses also have found an association between lesions in the insula and performance on verbal fluency [54], speech initiation, and motor planning [91, 92]. In addition, the insula has been shown to have strong connections to the LH prefrontal cortex, including the MFG and SMA [76], suggesting that in our patients, lesions affecting the LH insula and its connections with the LH SMA, the RH homologous frontal regions may result in recruitment of the RH insula and SMA for production processes. Alternatively, the RH SMA and insula may support these processes independent of lesioned tissue in the homologue LH regions.

Lastly, performance on the *spelling-to-dictation* measure was associated with GM volume in the right hippocampus and postcentral areas. Associations between performance on this task and GM volume in the RH hippocampal structures are in line with studies indicating a role of the hippocampus in healthy language learning [93–95], as well as with studies showing a positive correlation between treatment outcome and GM volume in the LH [96, 97] or bilateral hippocampus during recovery from stroke. Although—as previously acknowledged—the present study does not directly reflect “recruitment” of RH regions as part of recovery from aphasia, and the findings of a relation between GM volume and structures supporting healthy learning may not be coincidental. Further studies are necessary to investigate the role of the hippocampus as a structure supporting recovery in stroke-induced aphasia. Similarly, recruitment of the RH postcentral area may be implicated in recovery from aphasia. Previous neuroimaging studies in aphasic individuals have found RH postcentral gyrus activation across a variety of language tasks [24, 29].

Overall, our VBM results are inconsistent with those reported by Xing et al. [56]. Whereas Xing et al. found that GM volumes in right temporoparietal areas are related to speech production, but not comprehension, we found the opposite pattern. We found strong correlations between GM volumes in right temporal cortex and comprehension, but not production, and in the domain of production, we found that increased GM volume within the frontal region was associated with better production. It should be noted that the tasks used to test both comprehension and production differed across studies. To evaluate comprehension, Xing et al. [56] used data derived from WAB comprehension subtests and to evaluate production, spontaneous speech data and performance on a repetition task were used, whereas we used linguistically controlled, standardized comprehension and production tasks designed explicitly to elicit both comprehension and production of written and spoken words and sentences. We suggest that controlled tasks designed to measure specific language processes may better reflect neural recruitment patterns associated with recovery from aphasia.

To the extent that GM volume reflects functionality, the positive association between word comprehension and production ability and GM volume in the RH MTG and SMA, respectively, suggests that these regions may play a compensatory role in language recovery in aphasia. Although the precise mechanisms underlying RH GM volume are not completely understood, this finding is in keeping with one theory of language recovery—that RH regions are recruited to perform language functions when the LH is damaged. Notably, however, theories of language recovery suggest that RH compensation occurs in regions homologous to LH damaged regions. For example, in one study, Turkeltaub et al. [24] showed that people with lesions in the left IFG were more likely to recruit the right IFG than those without lesions in that area. Similarly, Buckner et al. [98] reported results of a single-stroke patient who showed activation in the right inferior prefrontal region during a word-stem completion task to compensate for lesioned tissue in the left frontal region, activated by healthy speakers. Also, see studies by Musso et al. and Perani et al., for similar patterns [18, 20]. However, the present data do not completely support this idea. Whereas, our patients with word comprehension impairments evinced lesions within the LH MTG, perhaps leading to recruitment of RH MTG, and our patients with sentence comprehension impairments evinced LH STG and MTG lesions, but no increases in GM volume were found in any RH regions. Further, our patients with production impairments did not present with LH SMA lesions but nevertheless showed increases in GM volume in the RH SMA, a nonhomologous region. One explanation for this latter finding is that the LH SMA is highly connected to regions within the LH that were damaged in our patients, perhaps leading to recruitment of its RH homologue.

In the absence of longitudinal data, however, we refrain from making strong claims regarding the relation between RH GM volume and recovery. Although RH regions may be recruited to support functions previously performed by LH regions, it is possible that RH recruitment may be maladaptive, as suggested by some repetitive transcranial magnetic stimulation studies (rTMS; see [6] for review). It also is possible that individual differences among participants before (rather than following) stroke may explain the RH GM volume differences we found between aphasic and healthy individuals. Although difficult to accomplish, longitudinal research in which individuals are tested prior to and following stroke could help to address this alternative hypothesis. Research examining GM volume in poststroke patients over time also will provide further insight into the extent to which GM changes are associated with language change. Indeed, the present data are part of a larger longitudinal study examining brain behavior changes associated with treatment (versus no treatment), and the results of which will be informative regarding neural recovery trajectories associated with improved language performance and yield a more comprehensive understanding of both structural and functional plasticity associated with language recovery in stroke aphasia.

## 5. Conclusion

This study examined the relation between the right hemisphere grey matter volume, left hemisphere lesion site, and both spoken and written comprehension and production of words and sentences in chronic stroke-induced aphasia. To the extent that RH grey matter volume reflects neural shifts associated with recovery from left hemisphere brain damage, our results indicate that right hemisphere regions, both homologous and nonhomologous to the left hemisphere lesioned regions, are recruited to support language, with unique recruitment patterns associated with language domain. Although further research is needed, the present findings have important implications for understanding poststroke neural reorganization.

## Figures and Tables

**Table 1:** Demographic data for aphasic and age-matched healthy participants.

	*N*	Age (yrs)	Gender	Education (yrs)	Time poststroke (months)
AM controls	40	58.9 (±11.8)	22F; 18M	15.6 (±2.4)	N/A
AM NU	11	54.8 (±8.2)	5F; 6M	16.4 (±1.6)	
AM BU	17	58.2 (±13.4)	8F; 9M	15.4 (±2.8)	
AM JHU	12	63.7 (±11.7)	9F; 3M	15.0 (±2.3)	

All aphasics	40	59.4 (±12.4)	14F; 26M	16.1 (±2.2)	57.2 (±52.3)
NU	11	49.0 (±8.0)	4F; 7M	16.9 (±2.1)	49.3 (±32.5)
BU	17	62.1 (±12.2)	5F; 12M	15.0 (±2.3)	44.3 (±40.7)
JHU	12	65.1 (±10.6)	5F; 7M	16.8 (±1.5)	82.7 (±72.7)

**Table 2:** Aphasic participants' scores on language measures.

Language domain		Test	All patients		BU		JHU		NU	
			Mean	SD	Mean	SD	Mean	SD	Mean	SD
	*Aphasia severity*	WABAQ	70.2	20.5	62.2	24.3	80.6	16.1	71.3	13.0
Comprehension	*Spoken word comprehension*	NNB AC	92.5	14.5	85.5	20.0	98.1	3.8	97.3	4.5
	*Word semantic association*	PALPA 51	64.0	20.1	54.1	23.7	73.3	16.2	69.7	13.6
	*Sentence comprehension*	NAVS SCT	71.4	17.3	71.0	19.6	78.9	17.9	63.9	8.5

Production	*Spoken word production*	NNB CN	70.5	29.7	59.6	37.4	76.6	21.6	80.7	17.8
	*Oral reading*	PALPA 35	65.1	34.3	56.4	41.3	68.5	28.0	76.0	25.9
	*Spelling-to-dictation*	PALPA 40	37.1	26.8	35.1	32.5	42.8	20.7	33.8	24.2
	*Sentence production*	NAVS SPPT	39.4	31.5	30.0	34.5	54.0	31.7	40.6	22.2

**Table 3:** Results of VLSM analyses by language measure.

Language measure	Test	LH regions (AAL)	Cluster size	Peak coordinates			*t* value	df	*p* (perm) correction	Benjamini-Hochberg correction	Effect size
				px	py	pz					
Spoken word comprehension	NNB AC	*Putamen*	949	−32	−17	0	5.15	35	0.026	0.068	0.656
		IFG									
		STG Rolandic operculum									
Word semantic association	PALPA51	*Putamen* Insula	796	−33	3	−9	4.49	34	0.015	0.068	0.61
		STG/MTG									
		Caudate									
Sentence comprehension	NAVS SCT	*MTG* STG	1040	−44	−23	0	4.98	35	0.029	0.068	0.644

*Note*. Table 3 summarizes regions where lesion volume was significantly associated with language performance in the comprehension domain. The results are presented at a threshold of *p* < 0.05, based on cluster size and the permutation method. In addition, the permutation-corrected *p* values were corrected for the total number of language measures examined (*n* = 7) using the Benjamini-Hochberg procedure. Significant peak regions are reported with the corresponding coordinates, *T* and *p* values, degrees of freedom, and effect sizes, as well as AAL regions included in the significant cluster; LH: left hemisphere; IFG: inferior frontal gyrus; STG: superior temporal gyrus; MTG: middle temporal gyrus.

**Table 4:** Results of VBM analyses by language measure.

Language Measure	Test	RH regions (AAL)	Cluster size	Peak coordinates			*t* value	df	FWE correction	Benjamini-Hochberg correction	Effect size
				px	py	pz					
Spoken word comprehension	NNB AC	Insula	1458	40.5	−4.5	−7.5	5.861	35	0.0000	0.0000	0.70
		MTG	732	55.5	−25.5	−4.5	4.646		0.0000	0.0001	0.62
Word semantic association	PALPA51			No sig. clusters							
Sentence comprehension	NAVS SCT			No sig. clusters							

Spoken word production	NNB CN	SMA	545	13.5	16.5	61.5	4.302	35	0.0001	0.0002	0.59
		Insula	267	40.5	−7.5	−7.5	4.106		0.0002	0.0003	0.57
Oral reading	PALPA35	SMA	502	13.5	15	61.5	4.549	34	0.0001	0.0001	0.62
		Pallidum	430	25.5	−1.5	−4.5	3.861		0.0005	0.0005	0.55
		Hippocampus	294	33	−28.5	−6	5.274		0.0000	0.0001	0.67
Spelling-to-dictation	PALPA40	Hippocampus	503	36	−1.5	−22.5	4.593	35	0.0001	0.0001	0.61
		Postcentral	258	49.5	−12	36	3.981		0.0003	0.0004	0.56
Sentence production	NAVS SPPT	SMA	275	13.5	15	58.5	4.625	34	0.0001	0.0001	0.62

*Note*. Table 4 summarizes regions where GM volume was significantly associated with language performance in both comprehension and production domains. The results are presented at a threshold of *p* < 0.05, based on *p* < 0.001 voxel-level threshold and a minimum cluster size (665–708 mm^3^) determined by an estimate of image smoothness. In addition, cluster *p* values were corrected for the total number of language measures examined (*n* = 7) using the Benjamini-Hochberg procedure. Significant peak regions are reported with corresponding coordinates, *T* and *p* values, degrees of freedom, and effect sizes; RH: right hemisphere; SMA: supplementary motor area; MTG: middle temporal gyrus.

## References

[1] Gainotti G. (2015). Contrasting opinions on the role of the right hemisphere in the recovery of language. A critical survey. *Aphasiology*.

[2] Kiran S. (2012). What is the nature of poststroke language recovery and reorganization?. *ISRN Neurology*.

[3] Price C. J. (2012). A review and synthesis of the first 20years of PET and fMRI studies of heard speech, spoken language and reading. *NeuroImage*.

[4] Thompson C. K., den Ouden D. B. (2008). Neuroimaging and recovery of language in aphasia. *Current Neurology and Neuroscience Reports*.

[5] Thompson C. K., Kielar A., Goldrick M., Ferreira V., Miozzo M. (2014). Neural bases of sentence processing: evidence from neurolinguistic and neuroimaging studies. *The Oxford Handbook of Language Production*.

[6] Turkeltaub P. (2015). Brain stimulation and the role of the right hemisphere in aphasia recovery. *Current Neurology and Neuroscience Reports*.

[7] Watila M. M., Balarabe S. A. (2015). Factors predicting post-stroke aphasia recovery. *Journal of the Neurological Sciences*.

[8] Fernandez B., Cardebat D., Demonet J. (2004). Functional MRI follow-up study of language processes in healthy subjects and during recovery in a case of aphasia. *Stroke*.

[9] Heiss W., Thiel A., Kessler J., Herholz K. (2003). Disturbance and recovery of language function: correlates in PET activation studies. *NeuroImage*.

[10] Ino T., Tokumoto K., Usami K., Kimura T., Hashimoto Y., Fukuyama H. (2008). Longitudinal fMRI study of reading in a patient with letter-by-letter reading. *Cortex*.

[11] Karbe H., Herholz K., Halber M., Heiss W. D. (1998). Collateral inhibition of transcallosal activity facilitates functional brain asymmetry. *Journal of Cerebral Blood Flow & Metabolism*.

[12] Saur D., Lange R., Baumgaertner A. (2006). Dynamics of language reorganization after stroke. *Brain*.

[13] Cao Y., Vikingstad E. M., George K. P., Johnson A. F., Welch K. M. A. (1999). Cortical language activation in stroke patients recovering from aphasia with functional MRI. *Stroke*.

[14] Fridriksson J., Bonilha L., Baker J., Mosen D., Rorden C. (2010). Activity in preserved left hemisphere regions predicts anomia severity in aphasia. *Cerebral Cortex*.

[15] Blasi V., Young A. C., Tansy A. P., Petersen S. E., Snyder A. Z., Corbetta M. (2002). Word retrieval learning modulates right frontal cortex in patients with left frontal damage. *Neuron*.

[16] Crinion J., Price C. J. (2005). Right anterior superior temporal activation predicts auditory sentence comprehension following aphasic stroke. *Brain*.

[17] Crosson B., Moore A. B., McGregor K. M. (2009). Regional changes in word-production laterality after a naming treatment designed to produce a rightward shift in frontal activity. *Brain and Language*.

[18] Musso M., Weiller C., Kiebel S., Müller S. P., Bülau P., Rijntjes M. (1999). Training-induced brain plasticity in aphasia. *Brain*.

[19] Ohyama M., Senda M., Kitamura S., Ishii K., Mishina M., Terashi A. (1996). Role of the nondominant hemisphere and undamaged area during word repetition in poststroke aphasics. A PET activation study. *Stroke*.

[20] Perani D., Cappa S. F., Tettamanti M. (2003). A fMRI study of word retrieval in aphasia. *Brain and Language*.

[21] Wan C. Y., Zheng X., Marchina S., Norton A., Schlaug G. (2014). Intensive therapy induces contralateral white matter changes in chronic stroke patients with Broca’s aphasia. *Brain and Language*.

[22] Weiller C., Isensee C., Rijntjes M. (1995). Recovery from Wernicke's aphasia: a positron emission tomographic study. *Annals of Neurology*.

[23] Winhuisin L., Thiel A., Schumacher B. (2005). Role of the contralateral inferior frontal gyrus in recovery of language function in poststroke aphasia: a combined repetitive transcranial magnetic stimulation and positron emission tomography study. *Stroke*.

[24] Turkeltaub P., Messing S., Norise C., Hamilton R. H. (2011). Are networks for residual language function and recovery consistent across aphasic patients?. *Neurology*.

[25] Breier J. I., Maher L. M., Novak B., Papanicolau A. C. (2006). Functional imaging before and after constraint-induced language therapy for aphasia using magnetoencephalography. *Neurocase*.

[26] Elkana O., Frost R., Kramer U., Ben-Bashat D., Schweiger A. (2013). Cerebral language reorganization in the chronic stage of recovery: a longitudinal fMRI study. *Cortex*.

[27] Fridriksson J., Morrow-Odom L., Moser D., Fridriksson A., Baylis G. (2006). Neural recruitment associated with anomia treatment in aphasia. *NeuroImage*.

[28] Fridriksson J., Moser D., Bonilha L. (2007). Neural correlates of phonological and semantic-based anomia treatment in aphasia. *Neuropsychologia*.

[29] Kiran S., Meier E. L., Kapse K. J., Glynn P. A. (2015). Changes in task-based effective connectivity in language networks following rehabilitation in post-stroke patients with aphasia. *Frontiers in Human Neuroscience*.

[30] Meinzer M., Obleser J., Flaisch T., Eulitz C., Rockstroh B. (2007). Recovery from aphasia as a function of language therapy in an early bilingual patient demonstrated by fMRI. *Neuropsychologia*.

[31] Thompson C. K., den Ouden D. B., Bonakdarpour B., Garibaldi K., Parrish T. B. (2010). Neural plasticity and treatment-induced recovery of sentence processing in agrammatism. *Neuropsychologia*.

[32] Raboyeau G., De Boissezon X., Marie N. (2008). Right hemisphere activation in recovery from aphasia lesion effect or function recruitment?. *Neurology*.

[33] Abel S., Weiller C., Huber W., Willmes K. (2014). Neural underpinnings for model-oriented therapy of aphasic word production. *Neuropsychologia*.

[34] Gold B. T., Kertesz A. (2000). Right hemisphere semantic processing of visual words in an aphasic patient: an fMRI study. *Brain and Language*.

[35] Thompson C. K., Riley E. A., Den Ouden D. B., Meltzer-Asscher A., Lukic S. (2013). Training verb argument structure production in agrammatic aphasia: behavioral and neural recovery patterns. *Cortex*.

[36] Allendorfer J. B., Kissela B. M., Holland S. K., Szaflarski J. P. (2012). Different patterns of language activation in post-stroke aphasia are detected by overt and covert versions of the verb generation task. *Medical Science Monitor: International Medical Journal of Experimental and Clinical Research*.

[37] Postman-Caucheteux W., Birn R., Pursley R. (2010). Single-trial fMRI shows contralesional activity linked to overt naming errors in chronic aphasic patients. *Journal of Cognitive Neuroscience*.

[38] Barwood C., Murdoch B., Whelan B. (2010). Improved language performance subsequent to low-frequency rTMS in patients with chronic non-fluent aphasia post-stroke. *European Journal of Neurology*.

[39] Hamilton R., Chrysikou E., Coslett H. B. (2011). Mechanisms of aphasia recovery after stroke and the role of noninvasive brain stimulation. *Brain and Language*.

[40] Martin P. I., Naeser M. A., Ho M. (2009). Overt naming fMRI pre- and post-TMS: two nonfluent aphasia patients, with and without improved naming post-TMS. *Brain and Language*.

[41] Naeser M., Martin P., Nicholas M. (2005). Improved picture naming in chronic aphasia after TMS to part of right Broca’s area: an open-protocol study. *Brain and Language*.

[42] Chieffo R., Ferrari F., Battista P. (2014). Excitatory deep transcranial magnetic stimulation with H-coil over the right homologous Broca’s region improves naming in chronic post-stroke aphasia. *Neurorehabilitation and Neural Repair*.

[43] Hartwigsen G., Saur D., Price C. J., Ulmer S., Baumgaertner A., Siebner H. R. (2013). Perturbation of the left inferior frontal gyrus triggers adaptive plasticity in the right homologous area during speech production. *PNAS*.

[44] Kakuda W., Abo M., Kaito N., Watanabe M., Senoo A. (2010). Functional MRI-based therapeutic rTMS strategy for aphasic stroke patients: a case series pilot study. *International Journal of Neuroscience*.

[45] Geranmayeh F., Brownsett S. L., Wise R. J. (2014). Task-induced brain activity in aphasic stroke patients: what is driving recovery?. *Brain*.

[46] van Oers C. A., Vink M., van Zandvoort M. J. (2010). Contribution of the left and right inferior frontal gyrus in recovery from aphasia. A functional MRI study in stroke patients with preserved hemodynamic responsiveness. *NeuroImage*.

[47] Baumgaertner A., Hartwigsen G., Siebner H. R. (2013). Right-hemispheric processing of non-linguistic word features: implications for mapping language recovery after stroke. *Human Brain Mapping*.

[48] Fein G., McGillivray S., Finn P. (2007). Older adults make less advantageous decisions than younger adults: cognitive and psychological correlates. *Journal of the International Neuropsychological Society*.

[49] Mungas D., Reed B. R., Jagust W. J. (2002). Volumetric MRI predicts rate of cognitive decline related to AD and cerebrovascular disease. *Neurology*.

[50] Zhang J., Meng L., Qin W., Liu N., Shi F. D., Yu C. (2014). Structural damage and functional reorganization in ipsilesional m1 in well-recovered patients with subcortical stroke. *Stroke*.

[51] Gauthier L. V., Taub E., Mark V. W., Barghi A., Uswatte G. (2012). Atrophy of spared gray matter tissue predicts poorer motor recovery and rehabilitation response in chronic stroke. *Stroke*.

[52] Schaechter J. D., Moore C. I., Connell B. D., Rosen B. R., Dijkhuizen R. N. (2006). Structural and functional plasticity in the somatosensory cortex of chronic stroke patients. *Brain*.

[53] Stebbins G. T., Nyenhuis D. L., Wang C. (2008). Gray matter atrophy in patients with ischemic stroke with cognitive impairment. *Stroke*.

[54] Ashburner J., Friston K. J. (2000). Voxel-based morphometry—the methods. *NeuroImage*.

[55] Bates E., Wilson S. M., Saygin A. P. (2003). Voxel-based lesion–symptom mapping. *Nature Neuroscience*.

[56] Xing S., Lacey E. H., Skipper-Kallal L. M. (2016). Right hemisphere grey matter structure and language outcomes in chronic left hemisphere stroke. *Brain*.

[57] Kertesz A. (2007). *Western Aphasia Battery (Revised)*.

[58] Thompson C. K., Weintraub S. (2014). *Northwestern Naming Battery (NNB)*.

[59] Kay J., Lesser R., Coltheart M. (1996). Psycholinguistic assessments of language processing in aphasia (PALPA): an introduction. *Aphasiology*.

[60] Thompson C. K. (2012). *Northwestern Assessment of Verbs and Sentences (NAVS)*.

[61] Tustison N. J., Avants B. B., Cook P. A. (2010). N4ITK: improved N3 bias correction. *IEEE Transactions on Medical Imaging*.

[62] Rorden C., Brett M. (2000). Stereotaxic display of brain lesions. *Behavioural Neurology*.

[63] Alpert K., Kogan A., Parrish T., Marcus D., Wang L. (2016). The Northwestern University Neuroimaging Data Archive (NUNDA). *NeuroImage*.

[64] Brett M., Anton J. L., Valabregue R., Poline J. B. (2002). Region of interest analysis using the MarsBar toolbox for SPM 99. *NeuroImage*.

[65] R Core Team (2015). *R: A Language and Environment for Statistical Computing*.

[66] Benjamini Y., Hochberg Y. (1995). Controlling the false discovery rate: a practical and powerful approach to multiple testing. *Journal of the Royal Statistical Society. Series B (Methodological)*.

[67] Cox R. W. (1996). AFNI: software for analysis and visualization of functional magnetic resonance neuroimages. *Computers and Biomedical Research*.

[68] Eklund A., Nichols T. E., Knutsson H. (2016). Cluster failure: why fMRI inferences for spatial extent have inflated false-positive rates. *Proceedings of the National Academy of Sciences*.

[69] Hart J., Gordon B. (1990). Delineation of single-word semantic comprehension deficits in aphasia, with anatomical correlation. *Annals of Neurology*.

[70] Dronkers N. F., Wilkins D. P., Van Valin R. D., Redfern B. B., Jaeger J. J. (2004). Lesion analysis of the brain areas involved in language comprehension. *Cognition*.

[71] Booth J. R., Burman D. D., Meyer J. R., Gitelman D. R., Parrish T. B., Mesulam M. M. (2002). Modality independence of word comprehension. *Human Brain Mapping*.

[72] Tyler L. K., Marslen-Wilson W. D., Stamatakis E. A. (2005). Differentiating lexical form, meaning, and structure in the neural language system. *Proceedings of the National Academy of Sciences of the United States of America*.

[73] Baker S. C., Frith C. D., Dolan R. J. (1997). The interaction between mood and cognitive function studied with PET. *Psychological Medicine*.

[74] Berlingeri M., Crepaldi D., Roberti R., Scialfa G., Luzzatti C., Paulesu E. (2008). Nouns and verbs in the brain: grammatical class and task specific effects as revealed by fMRI. *Cognitive Neuropsychology*.

[75] Kemeny S., Ye F. Q., Birn R., Braun A. R. (2005). Comparison of continuous overt speech fMRI using BOLD and arterial spin labeling. *Human Brain Mapping*.

[76] Ardila A., Bernal B., Rosselli M. (2014). Participation of the insula in language revisited: a meta-analytic connectivity study. *Journal of Neurolinguistics*.

[77] Friederici A. D. (2002). Towards a neural basis of auditory sentence processing. *Trends in Cognitive Sciences*.

[78] Rogalsky C., Hickok G. (2009). Selective attention to semantic and syntactic features modulates sentence processing networks in anterior temporal cortex. *Cerebral Cortex*.

[79] Friederici A. D., Kotz S. A., Scott S. K., Obleser J. (2010). Disentangling syntax and intelligibility in auditory language comprehension. *Human Brain Mapping*.

[80] Friederici A. D., Makuuchi M., Bahlmann J. (2009). The role of the posterior superior temporal cortex in sentence comprehension. *Neuroreport*.

[81] Mack J. E., Meltzer-Asscher A., Barbieri E., Thompson C. K. (2013). Neural correlates of processing passive sentences. *Brain Sciences*.

[82] Price C. J. (2010). The anatomy of language: a review of 100 fMRI studies published in 2009. *Annals of the new York Academy of Sciences*.

[83] Caplan D., Michaud J., Hufford R. (2015). Mechanisms underlying syntactic comprehension deficits in vascular aphasia: new evidence from self-paced listening. *Cognitive Neuropsychology*.

[84] Lukic S., Bonakdarpour B., den Ouden D. B., Price C., Thompson C. K. (2013). Neural mechanisms of verb and sentence production: a lesion-deficit study. *Procedia - Social and Behavioral Sciences*.

[85] Indefrey P., Levelt W. J. (2004). The spatial and temporal signatures of word production components. *Cognition*.

[86] Kawashima R., Okuda J., Umetsu A. (2000). Human cerebellum plays an important role in memory-timed finger movement: an fMRI study. *Journal of Neurophysiology*.

[87] Bohland J. W., Guenther F. H. (2006). An fMRI investigation of syllable sequence production. *NeuroImage*.

[88] Loucks T. M., Poletto C. J., Simonyan K., Reynolds C. L., Ludlow C. L. (2007). Human brain activation during phonation and exhalation: common volitional control for two upper airway functions. *NeuroImage*.

[89] Alario F. X., Chainay H., Lehericy S., Cohen L. (2006). The role of the supplementary motor area (SMA) in word production. *Brain Research*.

[90] Crosson B., Sadek J. R., Maron L. (2001). Relative shift in activity from medial to lateral frontal cortex during internally versus externally guided word generation. *Journal of Cognitive Neuroscience*.

[91] Dronkers N. F. (1996). A new brain region for coordinating speech articulation. *Nature*.

[92] Shuren J. (1993). Insula and aphasia. *Journal of Neurology*.

[93] Breitenstein C., Jansen A., Deppe M. (2005). Hippocampus activity differentiates good from poor learners of a novel lexicon. *NeuroImage*.

[94] Maguire E. A., Frith C. D. (2004). The brain network associated with acquiring semantic knowledge. *NeuroImage*.

[95] Opitz B., Friederici A. D. (2003). Interactions of the hippocampal system and the prefrontal cortex in learning language-like rules. *NeuroImage*.

[96] Meinzer M., Mohammadi S., Kugel H. (2010). Integrity of the hippocampus and surrounding white matter is correlated with language training success in aphasia. *NeuroImage*.

[97] Menke R. A., Scholz J., Miller K. L. (2009). MRI characteristics of the substantia nigra in Parkinson's disease: a combined quantitative T1 and DTI study. *NeuroImage*.

[98] Buckner R. L., Raichle M. E., Miezin F. M., Petersen S. E. (1996). Functional–anatomic studies of the recall of pictures and words from memory. *The Journal of Neuroscience*.

